# Groupness in Preverbal Infants: Proof of Concept

**DOI:** 10.3389/fpsyg.2017.00385

**Published:** 2017-03-16

**Authors:** Benjamin Sylvester Bradley, Michael Smithson

**Affiliations:** ^1^School of Psychology, Charles Sturt University, BathurstNSW, Australia; ^2^Department of Psychology, Australian National University, CanberraACT, Australia

**Keywords:** groupness, infant gaze, triadic behavior, socially directed behavior, dyadic program, attachment

## Abstract

Infant sociability is generally conceived in terms of dyadic capacities and behaviors. Recently, quantitative evidence has been published to support arguments that infants achieve a criterion for groupness: the capacity to interact simultaneously with two others. Such studies equate this capacity with alternating dyadic acts to the two other members of an interacting trio. Here we propose a stricter threefold criterion for infant groupness, of which the crux is whether the social behavior of an infant at time B is shown to be influenced by what two or more group-members were previously doing at time A. We test the viability of this conceptualization: (a) through its justification of the novel laboratory procedure of studying infant sociability in infant–peer quartets (rather than trios); and, (b) in an analysis of a pilot study of gaze-behavior recorded in 5-min interactions among two quartets of infants aged 6–9 months. We call this a ‘proof of concept’ because our aim is to show that infants *are capable of* groupness, when groupness is conceptualized in a supra-dyadic way—not that all infants will manifest it, nor that all conditions will produce it, nor that it is commonplace in infants’ everyday lives. We found that both quartets did achieve the minimum criterion of groupness that we propose: mutual gaze predicting coordinated gaze (where two babies, A and B, are looking at each other, and B is then looked at by C, and sometimes D) more strongly than the reverse. There was a significant absence of ‘parallel mutual gaze,’ where the four babies pair off. We conclude that, under specific conditions, preverbal infants can manifest supra-dyadic groupness. Infants’ capacities to exhibit groupness by 9 months of age, and the paucity of parallel mutual gaze in our data, run counter to the assumption that infant sociability, when in groups, is always generated by a dyadic program. Our conceptualization and demonstration of groupness in 8-month-olds thus opens a host of empirical, theoretical, and practical questions about the sociability and care of young babies.

## Introduction

Humans are group animals *par excellence*. Yet, theories of psychological development prevalently assume that group-level interaction emerges years after two-person relationships are established, if at all (Hay et al., 2011). This *dyadic assumption* reflects an enduring commitment in theories of socio-emotional growth. Thus a primary infant–adult attachment has long been held to establish or be generated by a “dyadic program” that underpins the development of all the infant’s subsequent social relationships (Bowlby, 1982, p. 378; Cassidy and Shaver, 2008). Here we present evidence that humans manifest group-level, *supra-*dyadic behavior—or ‘groupness’—during their first 9 months of life.

In social psychology, groupness implies an entity distinguishable from the characteristics of its members. This is held by Campbell (1958) to entail three features: the ‘common fate’ of a group’s members; their proximity; and their similarity. To test for groupness of this kind in infancy, we must examine whether a group of infants who are proximate (physically close) and similar (of the same age), exhibit ‘common fate.’ We argue common fate to be best assessed by gauging whether the behavior of two or more group-members at time A influences the behavior of at least one other group member at time B. Gaze is the most widely used behavior in studies of early sociability. Taking gaze as our target-behavior, we argue—from previous findings about groupness and about gaze—that groupness is more effectively conceived as responsiveness to two or more other group-members, than as actions simultaneously ‘aimed’ at more than one other. We road-test this conceptualization by applying it in quantitative analyses of gaze-behavior in two infant–peer quartets.

The most convincing studies of infantine groupness to date remain qualitative, showing that infants in trios can: simultaneously use different channels of communication (e.g., vocalizations, facial displays, and touch); interact with two others at once; respond to interactions between two others; and develop novel meanings during group-level interaction (Dunn and Kendrick, 1982; Fivaz-Depeursinge and Corboz-Warnery, 1999; Selby and Bradley, 2003). However, numerical data are now being marshaled as evidence for groupness in infant-including trios (McHale et al., 2008; Markova et al., 2010; Hay et al., 2011). It is this latter push we address here. First we demonstrate the short-comings of current observational criteria for ‘triadic’ behavior, arguing the need for a new, genuinely supra-dyadic conceptualization of groupness in infancy. We then test our proposed conceptualization: (a) through its justification of the novel laboratory procedure of studying infant sociability in infant–peer quartets (rather than trios); and, (b) in an analysis of gaze-behavior recorded in two quartets of 6–9 month-olds.

### Operationalizing Early Groupness

#### Measuring Infants’ Groupness

The commonest numerical index of infants’ sociability is “socially directed behavior” (SDB). An SDB is “any behavior accompanied by or immediately preceded or followed by looking at another person” (Mueller and Brenner, 1977, p. 856). As it is impossible to look at two people at once in an equilateral, triangular trio, SDBs only occur between two people. Thus Nadel and Tremblay-Leveau (1999, p. 205) reported with surprise that, in baby-baby-adult trios, 11-month-olds would sometimes direct their actions “to both” companions. They suggested redefining SDBs to accommodate “behavior directed at *two* persons,” by coding as *triadic* any behavior accompanied by “at least one discrete look at each of the two persons or to-and-fro gazing between both persons.” Subsequently, all researchers using statistical analyses to show that babies can interact with more than one person *at the same time* have equated simultaneous interaction with the *serial* production of dyadic behaviors aimed alternately at two different people over a given time-period (e.g., McHale et al., 2008, p. 452; Hay et al., 2011, p. 122).

The idea that an individual performing a *series* of *dyadic* acts might equate to interacting with two people *simultaneously* was first codified in Parke et al.’s (1979) analysis of “influence patterns” within family triads (e.g., father kisses mother who then nuzzles baby). Most quantitative studies of infants in trios adopt a serial, dyadic scheme of analysis akin to Parke et al.’s (1979). Thus Dunn and Kendrick’s (1982) analysis of the effects of a new sibling on an existing mother–child relationship is conceived dyadically. More recently, Ishikawa and Hay (2006) claim to have shown triadic interaction among toddlers entirely on grounds of a sequential dyadic analysis like Parke et al.’s (1979).

#### Triadic and Triangular

‘Triadic’ is the go-to concept when researching groupness in infancy. But researchers understand ‘triadic’ in two ways.

Most studies of babies’ *triadic* relations make the term refer to tasks which test whether a baby can follow an adult’s shift of attention to a nearby object. This person-person-object paradigm (cf. Heider, 1958) is particularly prevalent in studies of infant gaze-following and joint attention (e.g., Brooks and Meltzoff, 2005; Flom et al., 2007). It also shapes studies of social referencing. The person-person-object paradigm does not afford groupness.

Other studies use the term ‘triadic’ when arranging babies for interaction with two other people: the person-person-person paradigm—which we will distinguish from the above triadic studies as being *triangular*. Examples include a baby observed with both parents (Fivaz-Depeursinge and Corboz-Warnery, 1999); with a parent and a sibling (Dunn and Kendrick, 1982); with an adult and a same-age peer (Nadel and Tremblay-Leveau, 1999); or with two same-age peers (Markova et al., 2010). Triangular situations do afford groupness.

Adherence to triadic paradigms means that the social dynamics of infant looking also get *theorized* as dyadic. Thus, if we draw solely on such research, we must propose that looking in infant–peer groups results from a combination of cognitive rules such as: (1) If another baby shifts focus, follow their gaze. (2) If another baby looks at me, return their gaze. Both these rules are dyadic and have ample empirical warrant. The first rule formalizes the fact of infant gaze-following, and has been hypothesized to originate social cognition in infants (Brooks and Meltzoff, 2005). The second rule encases evidence that, from birth, infants prefer to look at front-on faces over alternatives (e.g., Gliga et al., 2009).

#### Gaze

Gaze is central to human social interaction, at all ages. The capacity to read others’ gaze-direction is found in several primate species, but is most developed in humans (Emery, 2000). Coding gaze is particularly important in the study of infant sociability (Beier and Spelke, 2012). This is for three reasons: it provides the simplest way to establish the directionality of an infant’s (social) acts; it is one of the first behaviors to come under intentional control; and gaze-changes are frequent, lending themselves to statistical analysis. For these reasons, the following discussion is dedicated to the study of gaze—though we believe our argument and method will prove applicable to other infant social behaviors.

As noted above, sequential interpretations of simultaneity in triangular settings are necessitated by the use of gaze to establish the directionality of infants’ social behavior (e.g., SDBs). However, this approach seriously over-simplifies infants’ visual capacities.

Firstly, studies of triangular interaction typically discount short looks, only counting those >1 s. Yet, in our data (see below), based on frame-by-frame analysis of high-quality zoomed-in video, 43% of infants’ looks at others were less than 1 s long and 19% were less than 0.5 s long (average look-length = 2.26 s; range: 0.12–15.28 s with one outlier at 40.04 s). We cannot assume *a priori* that these short looks are irrelevant to infant sociability.

Secondly, as noted already, the sequential measures of groupness employed in triangular research define simultaneous interaction as social behavior plus gaze-switching within a given time-period. The period may be anything from 3 s (McHale et al., 2008) to 30 s (Ishikawa and Hay, 2006). However, the average frequency of gaze-changes in the quartets of 6–9 month-olds we studied is one change every 3.7 s (see below). Given that the studies reviewed here involve three people positioned at such close quarters that gaze shifts *are likely* to be between the two people dominating the infant’s field-of-view, the fact that at least *some* purportedly ‘simultaneous’ interactions occur seems unremarkable.

Thirdly, humans have two mechanisms of vision: ambient (peripheral) and focal (central, macular). Ambient vision coordinates the whole field of space within which we respond and into which we can act. Ambient vision guides orientations of the head, postural changes, and locomotor displacements that alter the relationship between the body and spatial configurations of contours and surfaces, events and objects. Compared to focal vision, ambient visual awareness has far greater breadth (almost 180° laterally), low resolution for stationary features, low sensitivity for relative position, orientation or line, but high sensitivity to change in any of these attributes (Trevarthen, 1968, p. 328). In this sense, ambient vision affords *responsiveness* to a wide array of events. Thus ambient vision facilitates receptiveness. Focal vision, by contrast, has a very narrow field of view (around 13° laterally), being principally applied to one target-area which it swiftly samples by means of saccadic eye-movements (Yarbus, 1967). It thereby highlights a narrow field of identified objects, *into which it may guide voluntary action.* Focal vision is thus associated with agency.

No triangular studies have analyzed saccades. This is appropriate, because, prior to 10 months of age, infants in gaze-following tasks follow head-movements, but not eye-movements (Brooks and Meltzoff, 2005). This responsiveness to larger-scale postural changes is consistent with young infants’ use of ambient, not focal vision, in social situations. Nevertheless, researchers’ use of gaze as indicating infants’ social interests assumes their looking is solely focal: gaze being synonymous with deliberative ‘attention’ (cf. SDBs). Yet, were it to occur, group-level social interaction among infants *could not be guided by focal vision*, because the two (or more) people with whom an infant would be simultaneously interacting could not both/all fall within the narrow ∼13° field of his/her macular vision.

This recognition suggests that studying social *actions*, that are directed into the narrow field of an infant’s focal vision, when testing for groupness in infants, predisposes researchers to parse ‘group’ infant sociability as dyadic, in the manner criticized above. Researchers would do better to examine the *responsiveness* of infants to social events, as detected within their ambient visual field. The wide field detectable through ambient vision allows responsiveness to more than one other group-member at the same time. Which raises a new empirical question, one that is easier to answer than are questions framed in terms of an infant’s focal-gaze-directed actions toward other babies (as with SDBs)—given that such directionality is inevitably dyadic, not group-based, due to the narrow field of focal vision. This question is: can the synchronous (e.g., gaze) behavior of two or more persons at time A be used to predict what a baby, with whom they are apparently interacting, subsequently does (e.g., looks) at time B? Note that it is precisely this approach that proves fruitful in studying the coordination of collective behavior in group-living species like starlings (e.g., Bialek et al., 2012).

#### Conceptualizing Groupness for Research on Infants

Debates in social psychology about the distinction between dyadic and supra-dyadic behavior invoke various dimensions of groupness. However, any characteristic of an aggregate of individuals deemed to constitute a social group *must* assume a group is an entity distinguishable from the characteristics of its members. Contemporary approaches to groupness all treat of adults, and invoke criteria that are mediated verbally (Meneses et al., 2008). However, Campbell (1958), concerned about the empirical indeterminacy of psychologists’ conclusions about groupness, influentially proposed three *non-verbal* criteria for what he called the “entitativity” of groups, where entitativity meant the group’s “degree of having the nature of an entity, of having real existence,” or, of having a “completed boundary” (Campbell, 1958, pp. 17–18). These characteristics were: *common fate, similarity*, and *proximity*.

For Campbell (1958, p. 19), the paramount dimension of groupness was *common fate*, or the “covariability in time” of potential group-members’ behavior. If a coefficient of common fate were to identify a group, it had to tie together the behavior of at least three individuals over time such that the contemporaneous behavior of two (or more) members could be used to predict the behavior of a third. Beyond this, the *similarity* of members, and their *proximity* (“contemporaneous spatial contiguity”; p. 22), were less powerful components of groupness.

Following Campbell’s analysis, we propose that the minimum criterion for the observation of group-level interaction in preverbal infants would be: first, to constitute a potential group in which similarity and proximity were maximized; and secondly, to test whether the behavior of two or more infants, that is directed toward other members of their group, could be used to predict the behavior of one or more other group-members.

#### Proof of Concept

Theoretical questions about the social cognitive adaptations or acquisitions infants require to develop supra-dyadic competence cannot be answered until *a prior descriptive* question has been addressed: can infants participate in group-level social interaction and, if so, in what manner? Whether, they *are capable of* doing this is confirmable by a single rigorous observation. Hence, as a ‘proof of concept,’ the study reported below aims to test whether the supra-dyadic conceptualization of ‘groupness’ we have formulated above *can* be fruitfully applied to infants socializing under optimal conditions. We ask: in a group including infants where similarity and proximity are maximized, can the behavior of two or more infants, which is directed at other members of their group, be used to predict the behavior of other group-members? If the answer is affirmative, we will have shown that babies in groups *are capable of* supra-dyadic groupness, under specific and limited conditions. Our study will *not* have shown that all infants manifest groupness, nor that all conditions will produce it, nor that it is commonplace in infants’ everyday lives. But we will have shown how such possibilities could be tested. Hence, many further empirical (and theoretical) investigations will beckon.

## Materials and Methods

### Participants

Participants were eight healthy full-term infants recruited through posters put up in the surgeries of general practitioners in neighborhoods surrounding the Tavistock Institute of London. The study was approved through the London-Bloomsbury NHS Research Ethics Committee. All parents gave written informed consent prior to their child participating in the study. Parents were told that they could withdraw their child from the study at any time with our best wishes. Babies’ ages ranged from 6 months 21 days to 9 months 16 days, with mean of 8 months 7 days (*SD*: 31 days). Four babies, the ‘Pink group,’ attended together, comprising three girls and a boy. Ages in this group ranged from 6 months 21 days to 9 months 16 days, with a mean of 7 months 25 days (*SD*: 39 days). The other four babies, or ‘Checkered group,’ attended on another day, comprising two girls and two boys (gender was not controlled for, as we were not making between-group comparisons; and there was little reason to suppose that gender-balance would significantly affect groupness). Ages in this group ranged from 8 months 3 days to 9 months 5 days, with a mean of 8 months 19 days (*SD:* 19 days). Parents brought their babies to the Tavistock Institute by car or pre-paid taxi and played with their babies in a large playroom until the last member of the quartet arrived. They were then taken into the recording-studio.

Note that the two quartets analyzed here were recorded as part of a larger qualitative study. Nine quartets were recorded in all with a range of durations from 52 s to 6 min 37 s (Pink group), and a mean of 3 min 19 s (*SD*: 2 min). The Pink group and Checkered group (duration 5 min 9 s) were selected as optimal for this ‘proof of concept’ as lasting the longest.

### Design and Procedure

Our study was set up to analyze the influence of patterns of gaze involving two or more group-members on the subsequent gaze of a target member. We are assuming that such gaze-patterns will be detectable through infants’ ambient, if not focal, vision. To maximize member-similarity (Campbell’s second criterion), behavior was recorded in same-age infant–peer quartets, rather than the infant–adult trios used in all previous experimental tests. Asymmetries in communicative skill and power, knowledge, age, and body-size are minimized in all-infant groups, thus approximating an “ideal speech situation” more closely than in previous infants-plus-adults research (Selby and Bradley, 2003). Studying quartets rather than trios has the added advantage of ensuring that mutual gaze between two members does not necessarily entail the exclusion or isolation of the residual member(s), as is the case in trios.

**FIGURE 1:**
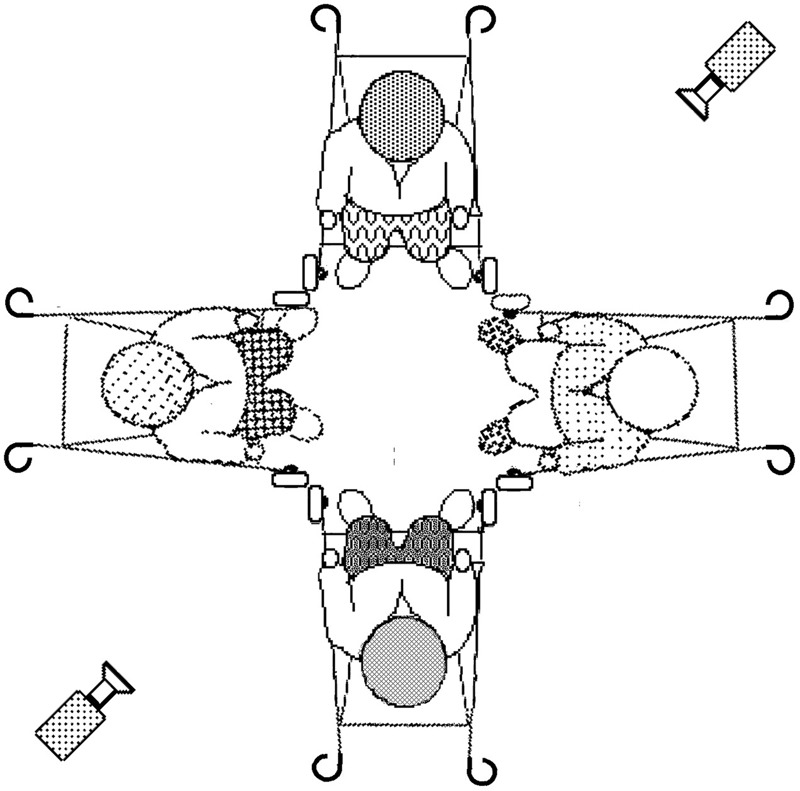
**Configuration of babies and cameras**.

To stabilize spatial symmetry and proximity (Campbell’s third criterion), each member of the quartet was secured in one of four immobilized push-chairs that had been configured to face one another in a tight square (**Figure 1**). The recording-studio was brightly and evenly lit. Two zoomed-in high-resolution digital video-cameras, with overlapping fields of view, simultaneously recorded the ensuing behavior of each baby’s whole body. The four immobilized push-chairs touched at their corners, the push-chairs’ foot-rests made a square with sides measuring 350 mm. The push-chairs’ harnesses were securely but loosely fastened to allow maximum freedom of movement of arms, legs, heads, and torsos. Each baby could easily touch the two neighboring babies foot-to-foot and, if they stretched, hand-to-hand. They could not touch each other hand-to-torso or head-to-head. They could touch their own feet with their hands and turn fully away from the other babies (e.g., to examine the push-chair). They could not touch the baby opposite.

As in previous work, parents watched from a near-by room through closed-circuit television (Selby and Bradley, 2003). If infants became distressed, parents or investigators halted recording and retrieved the babies from the recording studio.

While it might be thought more natural to have less apparatus, or for infants to disport themselves on the floor, pilot work with 6–9 month-olds showed that this was too chaotic to be ideal: at this age, infants vary considerably in mobility and postural control, may fall in awkward positions, or crawl off in unforeseen directions.

Once all four babies had been secured in their push-chairs, all adults vacated the studio to watch from an adjoining room by closed-circuit television. Recording continued until one or more babies expressed continuing frustration or the beginnings of distress.

### Coding

The first 5 min (7,500 frames at 25 frames per second) of interaction were coded for both quartets. The start, finish and target of every baby’s every look were coded frame by frame (i.e., every 1/25th of a second) by a coder blind to the hypotheses of this study. Each look was classified either as targeting one of the other three infants’ faces or as looking ‘elsewhere.’ Looks were deemed to commence with the first frame showing fixation of the target and to terminate with the last such frame. Of necessity, this meant that nearly all looks at babies’ faces were separated by, often very brief, looks ‘elsewhere,’ as a baby’s eyes transited between one baby’s face and another’s (unless the baby’s eyes were closed). Inter-observer reliabilities agreement levels for onset/offset of gazes were calculated on 35% of the data (298 onsets/offsets). The mean number of frames by which coders’ durations of agreed-upon gazes differed was 3.73. The correlation between the coder durations was 0.96. Out of 298 gazes, there were 2 gazes on which the coders disagreed about the target, and 9 gazes which one coder attributed to a target but the other did not.

The video-frame codes were converted into a synchronized frame-by-frame data-set. Statistical analyses were conducted in R 2.14 (R Development Core Team, 2011). These are fully described in Supplementary Materials. The second author (who conducted these analyses) had not viewed the videos and, in that sense, was ‘blinded’ with respect to the infants’ behavior.

### Data Analysis

**FIGURE 2:**
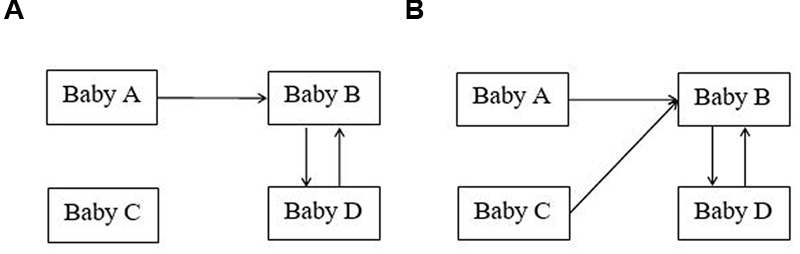
**Combinations of mutual and coordinated gaze. (A)** shows two babies and **(B)** shows three babies engaged in coordinated gaze.

Our study concerned a special case of supra-dyadic sociability, where there was evidence of a non-random co-occurrence of ‘mutual’ with ‘coordinated’ gaze-patterns (**Figure 2**). Coordinated gaze occurs when two (A and D look at B in **Figure 2A**) or three babies (A, C, and D look at B in **Figure 2B**) are simultaneously looking at the same target (another baby’s face). Mutual gaze occurs when two babies are looking at one another’s faces at the same time (i.e., B with D in **Figure 2**). Note that, given the group context of our study, our understanding of the relationships between mutual and coordinated gaze differs from the understanding typical of triadic studies, which involve mother, baby, and an object (e.g., Legerstee et al., 2007). In triadic studies, the statement that ‘mutual gaze predicts coordinated gaze’ must mean that ‘mutual gaze’ between mother and baby *is broken by* both mother and baby looking away to an object (‘coordinated gaze’). In our quartets, the statement ‘mutual gaze predicts coordinated gaze,’ means that mutual gaze between A and B *remains unbroken*, but *attracts the attention* of one or both of the other group members, C and D, such that at least two babies will then be looking at, say, B (coordinated gaze).

Our data were used to address two related questions: (1) Are occurrences of coordinated and mutual gaze correlated? (2) If the answer to the first question is “yes,” to what extent does one kind of gaze reliably predict or precede the other and over what time-spans? These questions address quite separate issues, with the first one being a synchronic matter and the second a diachronic matter. The first question concerns whether coordinated and mutual gaze occur simultaneously, whereas the second question is about whether coordinated and mutual gaze sequentially predict one another (and if so, which one more strongly predicts the later occurrence of the other).

Various sequences of event are compatible with such a prediction, including those that would suggest that babies are able to process and respond to what two or more other group-members are concurrently doing, although of course a causal relationship cannot be inferred from such evidence. For example, if two babies are looking at each other (B↔D), does this predict that other group-members (A and/or C) will look at one of them? Alternatively, if more than one baby is looking at a given peer (A and D or A and D and C at B), does this predictably lead to a mutual gaze (involving B)? Alternatively, mutual gaze between baby A and B, may be followed by coordinated gaze (a) of baby A and B at baby C, (b) of A and D at C, (c) C and B at A. The exact constellations of looking that underpin groupness are a topic for further research.

Our analysis began by converting the gaze onsets and offsets into a time-stream. The original codes had the form {*B_ij_, E_ij_, T_k_*}, where *B_ij_* denotes the onset of the jth look (e.g., it was the 17th look) for the ith baby (one of the four babies in the group) in number of frames from the start of the video (e.g., it began at frame 1000), *E_ij_* denotes its offset (the frame when it finished), and *T_k_* denotes the target (either another baby or “elsewhere”). The onsets of the first looks for the four babies were synchronized so that each baby’s time-stream data began simultaneously. The difference between each pair of starting- and ending-times, *E_ij_*–*B_ij_* = *D_ij_*, yielded the duration of each look in numbers of frames. The data for each baby therefore could be represented as a series of vectors of video frames denoting the gaze targets, with each gaze target *T_k_* repeated *D_ij_* times (durations of looks). The final version of the data was therefore a four-column matrix, each column corresponding to one of the babies, each of whose rows was a frame from the video, and each cell containing a letter denoting the target of that baby’s gaze.

Mutual gaze was operationalized as all frames in which a pair of babies was simultaneously looking at one another. Thus, the variable encoding the presence or absence of mutual gaze was binary, with each frame coded 1 if mutual gaze occurred and 0 if it did not. Coordinated gaze was operationalized as all frames in which two or more babies were simultaneously looking at the same baby. The variable representing the presence or absence of coordinated gaze also was binary, coded 1 if coordinated gaze was present and 0 if not. We recognize that this coding scheme may include various constellations of mutual and coordinated gaze that underpin “groupness” as we have conceptualized it. Hence, further research will be required to establish the mechanisms that may be involved in the relationship between these two kinds of gaze. Nevertheless, this scheme does enable an investigation into the extent to which the two types of gaze co-occur and, importantly, whether the occurrence of one predicts the onset of the other.

## Results

### Are Mutual and Coordinated Gaze Correlated?

To address our first question concerning the correlation of coordinated and mutual gaze, we began by identifying all frames involving mutual gaze and all frames involving coordinated gaze, cross-classifying them, and then estimating the conditional odds of one occurring given the occurrence of the other.

In the Pink group, we found that the odds of mutual gaze when there was coordinated gaze were 4.18 times higher than the odds of mutual gaze when there was no coordinated gaze. For the Checkered group, the odds of mutual gaze when there was coordinated gaze were 6.87 times higher than the odds of mutual gaze given no coordinated gaze (Supplementary Table S1).

There were two kinds of coordinated gaze: “two-gaze” in which two babies were looking at a third baby, and “three-gaze” in which three babies were looking at a fourth. In the Checkered group, 32.8% of coordinated gaze was three-gaze and in the Pink group 27.3% of coordinated gaze was three-gaze, so three-gaze was not uncommon. Moreover, the odds of mutual gaze when there was three-gaze was 7.00 times higher in the Pink group and 10.11 times higher in the Checkered group than when there was no coordinated gaze, whereas these odds-ratios were 3.44 and 5.73, respectively, for two-gaze versus no coordinated gaze. These descriptive statistics suggest that mutual and coordinated gaze are related, but they do not take autocorrelation into account.

A test that takes autocorrelation into account is a comparison of the observed transitions from one frame to the next with those expected by chance. Analyzing transitions is equivalent to differencing the series of frames, and differencing is a standard method in time series analysis for rendering a series stationary and thereby eliminating effects due to autocorrelation (Box et al., 1994). As shown in Supplementary Figure S1, both mutual and coordinated gaze series are autoregressive order 1 processes, and so differencing the series once is sufficient to create a stationary series from which the autocorrelation component has been removed.

Calling ‘simultaneous switches’ whenever two babies change the target of their gaze in the same frame, this test showed Pink and Checkered groups both exhibited more simultaneous switches to and from mutual and coordinated gaze (from **Table 1** and Supplementary Table S2: 20 + 13 = 33 and 23 + 18 = 41, respectively) than predicted by chance (2.46 and 2.68), and no frames in which mutual gaze switched on while coordinated gaze switched off, or vice-versa [χ^2^(1) = 409.45, *N* = 9515, *p* < 0.0001; and χ^2^(1) = 571.25, *N* = 7400, *p* < 0.0001].

**Table 1:** Coordinated and mutual gaze switching frequencies^∗^.

	Coordinated gaze					
Mutual	-1	0	1	-1	0	1
**Pink quartet**
-1	20	81	0	*1.23*	*98.54*	*1.23*
0	96	9114	103	*113.54*	*9085.93*	*113.54*
1	0	88	13	*1.23*	*98.54*	*1.23*
**Checkered quartet**
-1	23	84	0	*1.34*	*104.31*	*1.34*
0	103	7041	75	*90.31*	*7005.38*	*90.31*
1	0	89	18	*1.34*	*104.31*	*1.34*

*^∗^A -1 indicates that a gaze switched off, 0 indicates that its current state was maintained, and 1 indicates that it switched on. Expected frequencies are italicized.*

Note that this is a rather conservative test of whether these two types of gaze are related, because it does not incorporate reactive switching (e.g., where two babies’ commencement of mutual gaze triggers coordinated gaze from the remaining two babies). We are demonstrating here that when babies switch to the one kind of gaze behavior, they are more likely to be also switching to the other kind than would be predicted if these two kinds of gaze switched on and off independently or exclusively of one another. Note also that these findings are not intended as evidence for “groupness”; they simply provide evidence that mutual and coordinated gaze tend to co-occur, even when autocorrelation is taken into account. However, this is an essential result because the prediction analysis in the next section does not address the question of whether mutual and coordinated gaze are occurring simultaneously (e.g., it is possible that one could occur prior and/or subsequent to the other but not simultaneously).

### To What Extent Do Mutual Gaze and Coordinated Gaze Predict Each Other?

**FIGURE 3:**
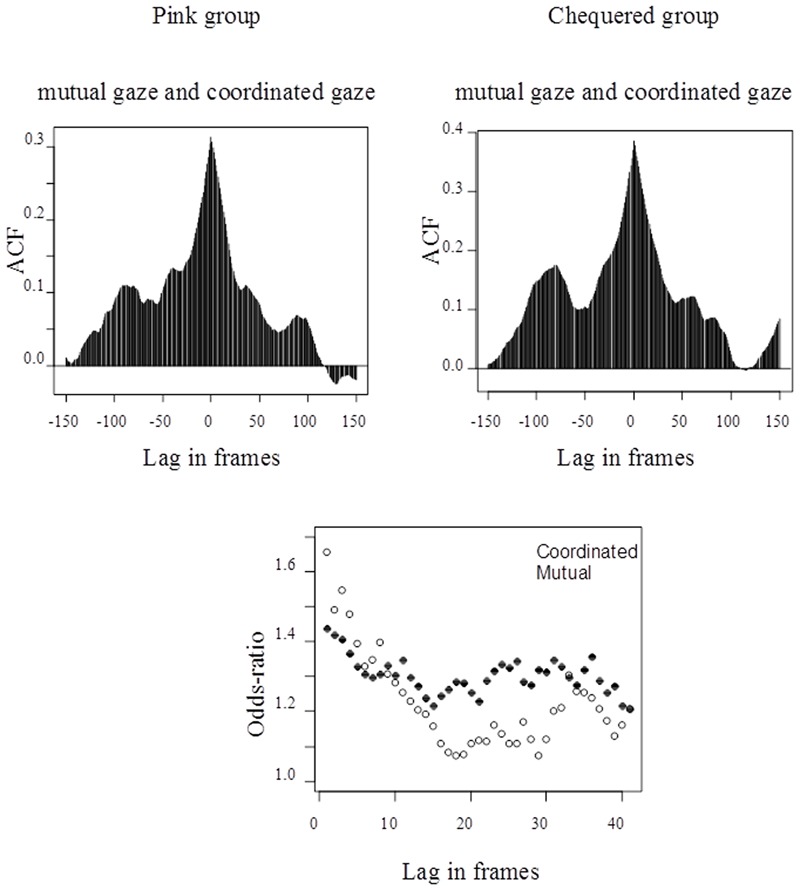
**Cross-correlation functions and logistic regression odds-ratios for mutual and coordinated gaze in both groups**.

This second question was examined via the cross-correlation function of the two kinds of gaze (see **Figure 2**), followed by logistic regressions with prediction in either direction at selected time-lags (taking autocorrelation into account). As shown in the Supplementary Materials, the partial autocorrelation functions for mutual and coordinated gaze demonstrated, in both groups, that both series were first-order auto-regression processes. The upper part of **Figure 3** (Supplementary Figure S2) shows the cross-correlation function for mutual and coordinated gaze, at lags within 150 frames (6 s) of the current frame. The negative lags are where mutual gaze was predicting coordinated gaze, and the positive lags are where coordinated gaze was predicting mutual gaze.

As can be seen from the two upper graphs, there was a mild tendency for mutual gaze more strongly to predict coordinated gaze than vice-versa until the lags approached 0, whereupon the correlation in either direction was equally strong. The implication is that, within approximately 100 frames (4 s), mutual and coordinated gaze predicted the onset of one another, and the relationship was moderately strong. This relationship was partly due to autocorrelation. However, the predictive relationships held up over lags of up to 39 frames (1.6 s) when autocorrelation was taken into account, as shown by logistic regressions (see Supplementary Table S4).

The bottom graph in **Figure 3** displays the odds-ratios from the logistic regressions (derived from Supplementary Table S4), with the legend denoting which kind of gaze is being predicted and the dashed line indicating the threshold for a significant odds-ratio. The predictor effects from both groups did not differ significantly (see Supplementary Table S3), so their data have been combined in the logistic regressions and in this graph. For example, in both groups the odds of mutual gaze occurring one frame later was 1.66 times higher when coordinated gaze occurred than when not, and the odds of coordinated gaze occurring one frame later was 1.44 times higher when mutual gaze occurred than when not.

Both groups displayed a markedly greater co-occurrence of mutual and coordinated gaze than would be expected by chance, and a moderately strong tendency for each type of gaze to predict the onset of the other, with coordinated gaze predicting mutual gaze up to 11 frames (0.44 s) in advance and mutual gaze predicting coordinated gaze up to 39 frames (1.56 s) in advance.

These findings provide evidence that at whom an infant looks in all-infant groups can be predicted by what two or more other group-members had previously been doing. This indicates that 6–9 month-olds may fulfill the minimum criterion for ‘groupness’ defined earlier.

## Discussion

Taken together, our results constitute *prima facie* evidence that infants *can*, at least sometimes, participate in group-level social interaction. As such, they demonstrate the efficacy of the conceptualization our study instantiates. The finding that mutual gaze predicts coordinated gaze more strongly, over a longer time period, than vice versa is not obviously expectable. Our evidence for this claim amounts to a variety of Granger (1969) causality, whereby one series over time predicts another to a stronger degree than vice-versa. While it might appear likely that coordinated gaze (e.g., A and D look at B) might lead to mutual gaze (e.g., B looks back at one of them, or at C), the finding that mutual gaze predicts coordinated gaze suggests that ‘pairing’ in a group attracts the attention of unpaired members. This seems counter-intuitive, if babies are born with a ‘shared dyadic program’ as attachment theory holds. Rather, the unpaired babies would themselves be expected to pair off with each other. But this kind of ‘parallel mutual gaze’ occurs only rarely, as we shortly will show.

But maybe group-level interaction is easily explicable in terms of the combination of simple ‘dyadic’ rules for looking? Two such rules, discussed previously, can be tested against our data, namely: (1) If another baby in the group shifts focus, follow their gaze; (2) If another baby looks at me, return their gaze. However, while Rule 1 adequately formalizes findings from “gaze following” experiments, it seriously under-determines an infant’s options in a four-infant quartet. There are three babies’ gazes to follow, so which one shall a baby choose?

Had these two looking rules obtained in our quartets, we would expect to have found plentiful occasions when mutual gaze switches on while coordinated gaze simultaneously switches off, and vice-versa. However, the pattern of co-occurring switches in both quartets is remarkable because the only non-zero frequencies are for switching off and switching on *both* mutual *and* coordinated gaze in the same frame. In neither quartet are there any frames in which mutual gaze switches on while coordinated gaze simultaneously switches off, or vice-versa (**Table 1** above). This is fairly strong evidence for a positive relationship between mutual and coordinated gaze. As such, our data depart significantly from the pattern predicted by the ‘two rule’ hypothesis under discussion.

The ‘two-rule hypothesis’ would also predict plenty of parallel mutual gaze cases, as just argued. Yet we found a near absence of ‘parallel mutual gaze.’ In the Pink quartet there are no frames in which this occurs, and in the Checkered quartet there are just nine such frames, i.e., a total duration of 0.36 s.

The nearly complete absence of parallel mutual gaze noted in refuting the ‘two rule’ hypothesis has an additional implication. While our data suggest that infants *can* be influenced by more than one other group-member simultaneously, infants might still *prefer* to interact dyadically when possible. But, far from ‘falling naturally’ into dyads, the infants in our study proved less rather than more interested in pairing off themselves when other group-members visually pair.

By demonstrating that groupness, as we conceive it, *can* occur in quartets of infants aged around 8 months of age, we open up many new empirical questions—as well as providing a refined conceptualization of groupness, and a new analytic approach, by which such questions can be answered. As already mentioned, there are various configurations of transition in who looks at whom which could underlie our findings that coordinated and mutual gaze predict each other. Investigating this issue would require that analyses noted which particular babies were involved in mutual and coordinated gaze at all times. Another obvious extension of our research is to ask: what kinds of multi-participant or single-participant interactive events are associated with early groupness? Existing qualitative and quantitative research on infant–peer and infant–adult trios suggests a wide range of possible triggers and accompaniments, for example: play between group-members; imitation by group-members of a peer’s action; and focused, friendly interaction between two group-members (e.g., Selby and Bradley, 2003; Fivaz-Depeursinge et al., 2005; Fivaz-Depeursinge and Favez, 2006). Amongst these, perhaps the most obvious is: how do group-members respond to noisy vocalization, and, in particular, distress? Here, a study by Liddle et al. (2015) is illustrative, although it did not employ the concept of groupness proved here. Liddle et al. (2015) showed that 8-month-olds’ distress in infant–peer trios always elicited gaze from other group members—plus, albeit to a lesser extent, non-distressed social behavior: responses that had the effect of significantly reducing that distress. Presumably, if future studies show that *non-distressed* vocalization also provokes and/or accompanies groupness, this would be germane to understanding the development of group communication, and of language. Beyond this there is a host of other questions that can also now be tackled—having to do, for example, with such questions, as: the role of individual differences in groupness, including gender, temperament, social background, and age; or the place of groupness in early child care (e.g., Sumsion et al., 2016).

## Conclusion

The idea that human social groups are more and other than the sum of their members’ individual characteristics—that groups have a real entitative existence *qua* groups*—*has been fundamental to social psychology from its inception (Meneses et al., 2008). Over the last 20 years, claims that infants manifest groupness have begun to occur in developmental psychology. While there is strong qualitative evidence for supra-dyadic interaction in preverbal infants, we have argued that the quantitative evidence previously collected to support the conclusion that babies can simultaneously interact with more than one person is seriously flawed, being: (a) dyadic; and (b) sequential, not simultaneous.

This paper clarifies what needs to have been observed to suggest that preverbal infants are capable of manifesting groupness. Our conceptual analysis of triadic-ness, groupness, and infants’ visual capacities, suggested a new minimum criterion for the statistical demonstration of supra-dyadic interaction in infancy. This is based on an infant’s *responsiveness* to others (rather than on the multi-directedness of the infant’s own social actions): something likely to be mediated by infants’ ambient vision, not their focal vision. Our criterion requires evidence that a baby’s behavior at Time B can be predicted from what two or more other group-members were simultaneously doing at a previous Time A. We tested the viability of this concept by applying it to the analysis of visual behavior in two infant–peer quartets.

Our results show that babies *can* achieve the minimum criterion of group-level interaction that we have proposed. While we have analyzed only two quartets, comprising eight babies, the effective ‘sample size’ in our analyses of gaze-behavior comprises 100s of gazes. Further, the fact that not just one but both the quartets we tested achieved this criterion strengthens the claims to real-world applicability of our conceptualization of groupness in infancy. Moreover, some details of our results suggest that the phenomena we have observed will not easily be explicable in terms of dyadic looking rules or the shared dyadic program said to underpin infant–adult attachment.

It should be emphasized that our results show only that infants *are capable of* supra-dyadic groupness. We make no claim to generalize these results to all babies, or to all conditions, or all infants’ social behaviors, or to the everyday circumstances in which babies live. We have only shown that—and how—a supra-dyadic concept of groupness can be applied in empirically investigations of infant sociability. Yet, if our approach were adopted in larger studies, across more diverse conditions, with more varied types of baby, recording more varied behaviors than just gaze, they would test new and significant questions for the understanding of socio-emotional development. For example, how does early experience of group interaction—in child care or larger families, for example—affect later social and emotional development?

In future, it will be possible to refine the understanding of infants’ capacity for groupness that has been presented here. For example, using Campbell’s (1958) understanding of groupness to refine the study-design we have presented, could lead to tests of the degree to which *dis*similarities between group members might affect group-level social interaction involving babies (e.g., gender, age, socio-economic background, family type, attachment classification, degree of familiarity, depressed versus non-depressed mother). In short, should infants be widely found able to engage in group-level interaction, the theoretical, empirical, and practical consequences would clearly be extensive, both for the conceptualization and study of human sociability and development, and for the optimal promotion of infants’ care and welfare. Our study has shown that there is an effective way to take up these new opportunities for the empirical study of supra-dyadic groupness in the first year of life.

## Author Contributions

BB conceived the study, reviewed the literature, obtained the grants, supervised data-collection, analyzed the videos and supervised the collection of inter observer reliability data. He also wrote the first draft of the article, bar the detailed description of data-analysis. MS conceived and conducted all mathematical analyses and data-presentations, both for the main article and the supplementary information. He also wrote the bulk of the section of the paper describing the data analysis and in the supplementary information. MS and BB then co-edited the final draft.

## Conflict of Interest Statement

The authors declare that the research was conducted in the absence of any commercial or financial relationships that could be construed as a potential conflict of interest.
