# Supplementary material for: Groupness in Preverbal Infants: Proof of Concept
Source: Front Psychol. 2017 Mar 16;8:385. doi: 10.3389/fpsyg.2017.00385 (PMC5352679; doi:10.3389/fpsyg.2017.00385)
Supplement: Supplementary file 1 [file Presentation_1.PDF]

Supplementary Methods: Data Analysis

This paper finds that looking behaviour in infant-peer quartets shows a strong predictive link between mutual gaze (reciprocal looking involving two infants) and coordinated gaze (where babies look simultaneously at the same target-baby), and a paucity of parallel mutual gaze, where the quartet divides into two pairs.

Pre-Processing

We converted the gaze onsets and offsets into a time-stream. The original codes had the form $\{B_{ij}, E_{ij}, T_k\}$, where B_{ij} denotes the onset of the jth look for the ith baby in number of frames from the start of the video, E_{ij} denotes its offset, and T_k denotes the target (either another baby or "elsewhere"). The onsets of the first looks for the four babies were synchronized so that each baby's time-stream data began simultaneously. The difference between each pair of starting- and ending-times, $E_{ij} - B_{ij} = D_{ij}$, yielded the number of frames' duration of each look. The data for each baby therefore could be represented as a series of vectors of video frames denoting the gaze targets, each T_k repeated D_{ij} times. The final version of the data was therefore a four-column matrix, each column corresponding to one of the babies, each of whose rows was a frame from the video, and each cell containing a letter denoting the target of that baby's gaze.

The time-series was used to generate an animation of the babies' gaze behaviours for each quartet, which was coded in R 2.14[25] (The files for running both animations are available at https://dl.dropbox.com/u/1857674/Babies/animate.htm). Both animations strongly indicated a prevalence of co-occurring mutual gaze and coordinated gaze, which underpinned the results reported in this paper. Mutual gaze was operationalized as all frames in which a pair of babies was simultaneously looking at one another. Thus, the variable encoding the presence or absence

of mutual gaze was binary, with each frame coded 1 if mutual gaze occurred and 0 if it did not. Coordinated gaze was operationalized as all frames in which two or more babies were simultaneously looking at the same baby. The variable representing the presence or absence of coordinated gaze also was binary, coded 1 if coordinated gaze was present and 0 if not.

Autocorrelation

Clearly frame-by-frame gaze behavior is likely to be positively auto-correlated, in the sense that if baby A is looking at baby B in one frame, A probably will be looking at B in the next frame. A precondition for effective statistical modelling of gaze behavior therefore is determining its autocorrelation structure. A standard method of doing so begins by inspecting the autocorrelation function (ACF) and partial autocorrelation function (PACF). The latter is a graph of the partial correlation between gaze target in the current frame and gaze target in an earlier frame, with the correlation with gaze in frames preceding the earlier frame partialled out. Figure S1 shows the PACFs for mutual and coordinated gaze for the Pink group (top half of the figure) and the Chequered group (bottom half). All four PACFs are very similar and strongly indicate an autoregressive order 1 (AR(1)) process, whereby gaze in the current frame is predicted by gaze in the preceding frame. This is indicated by the single high spike at lag =1, as can be seen in Figure S1 for mutual and coordinated gaze in both quartets. None of the later lags (out to about 2000 frames) show any significant or substantial partial correlation with the current frame. The subsequent analyses of these data take this autocorrelation structure into account.

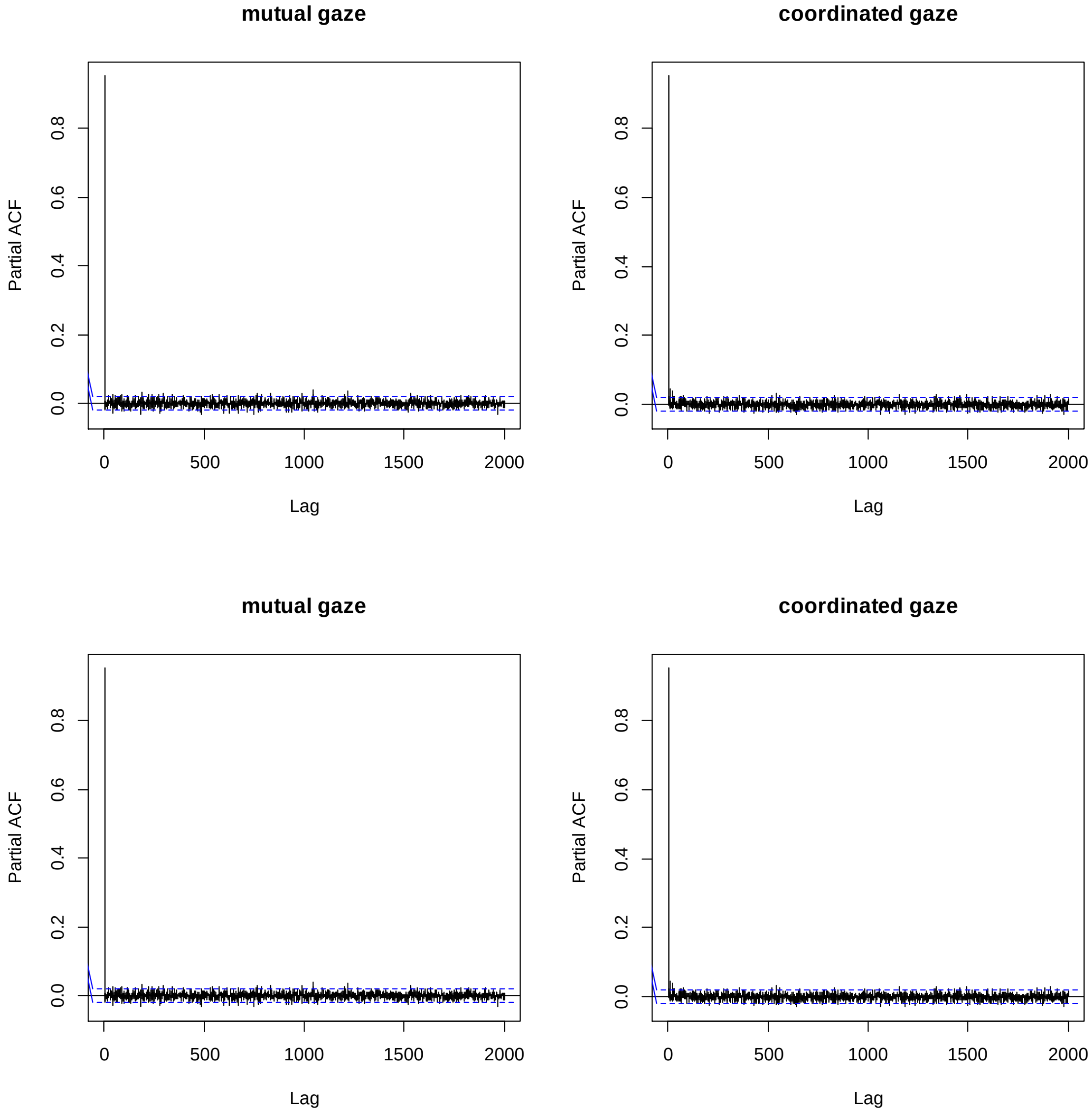

Figure S1. ACFs for mutual and coordinated gaze for two quartets, Pink (top half) and Chequered (bottom half)

Co-occurrence of mutual and coordinated gaze

Table S1 display the crosstabulations of coordinated and mutual gaze co-occurrences for both quartets. In the Pink quartet, the odds of mutual gaze given coordinated gaze are 2392/2674 = 0.895 whereas the odds of mutual gaze when coordinated gaze is absent are 784/3666 = 0.219, so the odds of mutual gaze given coordinated gaze 4.18 times higher than the odds of mutual gaze given no coordinated gaze. Likewise, in the Chequered quartet, the odds of mutual gaze given coordinated gaze are 2757/2148 = 1.284 whereas the odds of mutual gaze when coordinated gaze is absent are 393/2103 = 0.187, so the odds of mutual gaze given coordinated gaze 6.87 times higher than the odds of mutual gaze given no coordinated gaze.

Table S1. Coordinated and mutual gaze co-occurrence frequencies

Pink quartet

	coordinated gaze	
mutual	absent	present
absent	3666	2674
present	784	2392

Chequered quartet

	coordinated gaze	
mutual	absent	present
absent	2103	2148
present	393	2757

Both mutual and coordinated gaze appear to be AR(1) processes. We present a test of co-occurrence here that takes this into account, by differencing both gaze series. Differencing is a

standard method in time series analysis for rendering a series stationary and thereby eliminating effects due to autocorrelation (Box, Jensen, & Reinsel 1994), and differencing once is sufficient for an AR(1) process.The differencing is achieved by dummy-coding both mutual and coordinated gaze, each getting a value of 1 in a frame when it is present and 0 when it is absent. Taking the differences between successive frames yields -1 (switching from presence to absence), 0 (no switching), or 1 (switching from absence to presence).

As shown in Table S2, switching does not occur nearly as often as no switching, and we should expect the co-occurrence of switching to be quite rare. Indeed, the expected number of co-occurring switches for the Pink quartet is 4*1.23 = 4.92, and for the Chequered quartet it is 4*1.34 = 5.36. However, the total observed number of co-occurring switches in the Pink quartet is 20 + 0 + 0 + 13 = 33, and for the Chequered quartet it is 23 + 0 + 0 + 18 = 41. These results are statistically significant ($\chi^2(1) = 409.45$, $N = 9515$, $p < .0001$; and $\chi^2(1) = 571.25$, $N = 7400$, $p < .0001$).

Moreover, the pattern of co-occurring switches in both quartets is remarkable because the only nonzero frequencies are for switching off and switching on both mutual and coordinated gaze in the same frame. In neither quartet are there any frames in which mutual gaze switches on while coordinated gaze switches off, or vice-versa. This is fairly strong evidence for a positive relationship between mutual and coordinated gaze.

Table S2. Coordinated and mutual gaze switching frequencies*

	Pink quartet					
	coordinated gaze					
mutual	-1	0	1	-1	0	1
-1	20	81	0	*1.23*	*98.54*	*1.23*
0	96	9114	103	*113.54*	*9085.93*	*113.54*
1	0	88	13	*1.23*	*98.54*	*1.23*

	Chequered quartet					
	coordinated gaze					
mutual	-1	0	1	-1	0	1
-1	23	84	0	*1.34*	*104.31*	*1.34*
0	103	7041	75	*90.31*	*7005.38*	*90.31*
1	0	89	18	*1.34*	*104.31*	*1.34*

* Expected frequencies are italicized.

The co-occurrence relationship between mutual and coordinated gaze is further buttressed by the absence of parallel mutual gaze. Parallel mutual gaze occurs when two pairs of babies simultaneously engage in mutual gaze. In the Pink quartet there are no frames in which this occurs, and in the Chequered quartet there are just 9 such frames, i.e., a total duration of 9/25 = 0.36 seconds.

Cross-correlation function

The cross-correlation function (CCF) for each quartet was examined over 2000 lags initially, and the results are graphed in the upper part of Figure S2. There is a distinct region of lags in which the CCF is positive for both quartets, and the lower part of Figure S2 focuses on that

region. The dashed horizontal lines in both sets of graphs indicate the bounds beyond which correlations are significant, so it can be seen that within approximately 100 lags the CCFs for both quartets show significant positive correlations.

Pink quartet Chequered quartet

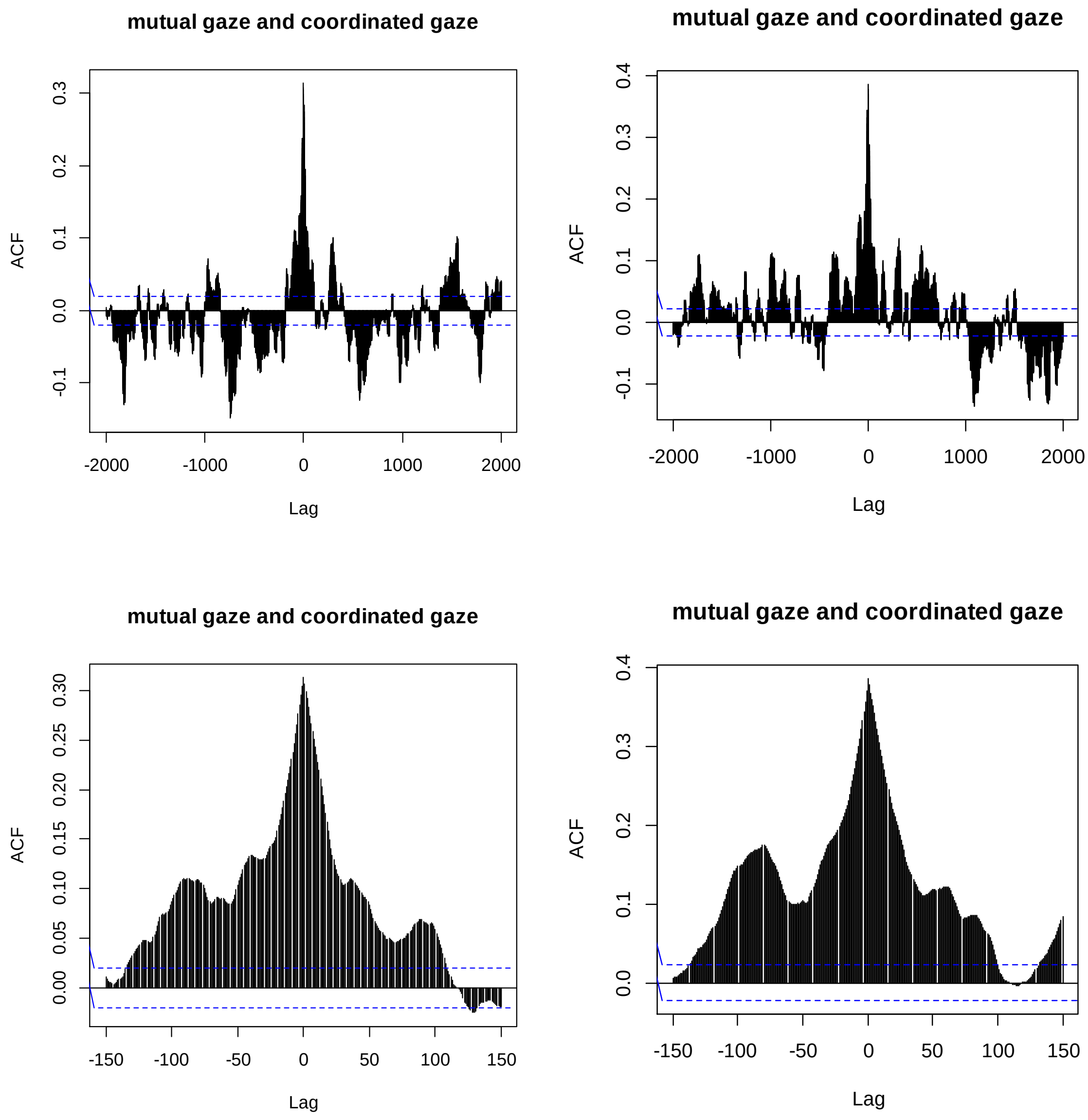

Figure S2. CCFs for mutual and coordinated gaze for two quartets, Pink (top half) and Chequered (bottom half)

In the negative lags mutual gaze is predicting coordinated gaze, and in the positive lags coordinated gaze is predicting mutual gaze. Thus, it can be seen that to a modest degree mutual gaze appears to predict coordinated gaze more strongly than vice-versa. However, the CCF is partly due to autocorrelation and as will become clear in the next section, this tendency does not hold up when autocorrelation is taken into account.

Prediction controlling for autocorrelation

Two sets of logistic regressions were conducted on the mutual gaze and coordinated gaze data, one for predicting mutual gaze and the other for predicting coordinated gaze. Both logistic regression models incorporated autocorrelation by including the dependent variable lagged by one frame as a predictor, with the other predictor then lagged by varying numbers of frames to ascertain its conditionally independent predictive contribution to the model.

The predictor effects do not significantly differ between the two quartets because when the predictor-by-quartet interaction terms are included in the model they are not significant at any lags. Evidence for this is presented in Table S3, where the z statistics for these interaction terms and their significance levels are displayed for lags ranging from 1 to 15 frames. The interaction terms for the model predicting mutual gaze occupy the left half of the table, and the interaction terms for the model predicting coordinated gaze occupy the right half.

The final version of the logistic regression models for mutual and coordinated gaze include main-effects terms for the lagged dependent variable, quartet, and predictor, plus a lagged dependent variable-by-quartet interaction term. The latter is consistently significant for the model predicting coordinated gaze but not so for the model predicting mutual gaze; it is included simply to ensure that the autocorrelation structure of both groups is fully accounted for.

Table S3. Predictor interaction-term *z* statistics at selected lags

	mutual gaze		coordinated gaze	
lag	*z*	*p*	*z*	*p*
1	-1.440	.150	0.283	.777
3	-1.429	.153	0.931	.352
5	-1.455	.146	1.251	.211
7	-1.328	.184	1.291	.198
9	-1.330	.184	0.747	.455
11	-1.349	.177	0.799	.424
13	-1.333	.183	0.761	.447
15	-1.397	.162	0.808	.419

Table S4 shows the regression coefficients for the predictors at selected lags. The significance levels in this table demonstrate that mutual gaze is significantly predicted by coordinated gaze up to a lag of 11 frames and occasionally thereafter, while coordinated gaze is predicted by mutual gaze for lags up to nearly 40 frames (a lag of 39 yields the last significant coefficient). The lower part of Figure 3 in the paper graphs the odds-ratios predicted by these logistic regressions.

Table S4. Logistic regression coefficients for main effect of predictor at selected lags

	mutual gaze				coordinated gaze			
lag	β	*s.e.*	*z*	*p*	β	*s.e.*	*z*	*p*
1	0.506	0.111	4.568	< .0005	0.363	0.106	3.438	.0006
2	0.400	0.111	3.603	< .0005	0.351	0.105	3.343	.0008
3	0.437	0.110	3.955	< .0005	0.342	0.105	3.267	.0011

4	0.392	0.110	3.549	< .0005	0.313	0.104	2.996	.0027
5	0.333	0.110	3.020	.0025	0.284	0.104	2.729	.0063
6	0.286	0.110	2.589	.0096	0.267	0.104	2.573	.0101
7	0.298	0.110	2.709	.0068	0.262	0.104	2.528	.0115
8	0.336	0.109	3.067	.0022	0.269	0.103	2.603	.0092
9	0.269	0.110	2.454	.0141	0.288	0.103	2.799	.0051
10	0.248	0.109	2.263	.0236	0.267	0.103	2.600	.0093
11	0.227	0.109	2.075	.0380	0.299	0.102	2.923	.0035
12	0.206	0.109	1.888	.0591	0.261	0.102	2.551	.0107
13	0.186	0.109	1.702	.0888	0.243	0.102	2.376	.0175
14	0.177	0.109	1.627	.1038	0.214	0.102	2.098	.0359
15	0.146	0.109	1.338	.1808	0.195	0.102	1.916	.0553
20	0.103	0.108	0.957	.3386	0.228	0.101	2.250	.0244
25	0.103	0.107	0.957	.3385	0.283	0.101	2.804	.0051
30	0.114	0.107	1.071	.2840	0.273	0.101	2.716	.0066
35	0.227	0.106	2.137	.0326	0.279	0.100	2.776	.0055
40	0.148	0.106	1.394	.1634	0.196	0.100	1.951	.0511
45	0.191	0.106	1.793	.0729	0.129	0.100	1.282	.1997
